# Supplementary material for: Conditional chemoconnectomics (cCCTomics) as a strategy for efficient and conditional targeting of chemical transmission
Source: eLife. 2024 Apr 30;12:RP91927. doi: 10.7554/eLife.91927 (PMC11060718; doi:10.7554/eLife.91927)
Supplement: Figure 3—source data 3. [file elife-91927-fig3-data3.zip › Figure 3 source data3 part 2.zip!/Figure 3-Source data 3-part2/1025-13-CRISPResso_Report_ack040l3.zip!/CRISPResso_on_1025_15/2a.Nucleotide_percentage_quilt.pdf]

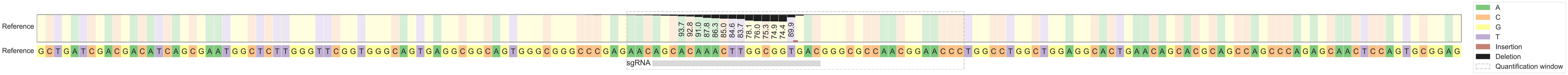
Reference
93.7
92.8
91.0
87.8
86.3
85.0
84.6
83.7
78.1
76.0
75.3
74.9
74.4
89.9
Reference
GCTGATCGACGACATCAGCGAATGGCTCTTGGGTTCGGTGGGCAGTGAGGCGGCAGTGGGCGGGCCCGAGAACAGCACAAACTTGGCGGTGACGGGCGCCAACGGAACCCTGGCCTGGCTGGAGGCACTGAACAGCACGCAGCCAGCCCAGAGCAACTCCAGTGCGGAG
sgRNA
A
C
G
T
Insertion
Deletion
Quantification window
